# Prototype Gluten-Free Breads from Processed Durum Wheat: Use of Monovarietal Flours and Implications for Gluten Detoxification Strategies

**DOI:** 10.3390/nu12123824

**Published:** 2020-12-14

**Authors:** Rosa Pilolli, Maria De Angelis, Antonella Lamonaca, Elisabetta De Angelis, Carlo Giuseppe Rizzello, Sonya Siragusa, Agata Gadaleta, Gianfranco Mamone, Linda Monaci

**Affiliations:** 1 Institute of Sciences of Food Production, CNR-ISPA, 70126 Bari, Italy; antonella.lamonaca17@libero.it (A.L.); elisabetta.deangelis@ispa.cnr.it (E.D.A.); linda.monaci@ispa.cnr.it (L.M.); 2 Department of Soil, Plant and Food Science, Università degli Studi di Bari Aldo Moro, 70126 Bari, Italy; maria.deangelis@uniba.it (M.D.A.); carlogiuseppe.rizzello@uniba.it (C.G.R.); sonya.siragusa@uniba.it (S.S.); 3 Department of Agricultural and Environmental Sciences, Università degli Studi di Bari Aldo Moro, 70126 Bari, Italy; agata.gadaleta@uniba.it; 4 Institute of Food Sciences, CNR-ISA, 83100 Avellino, Italy; gianfranco.mamone@isa.cnr.it

**Keywords:** gluten-free, detoxification strategies, sourdough, celiac disease, epitopes, in-vitro simulated human gastroduodenal digestion

## Abstract

In this investigation, we reported the production of prototype breads from the processed flours of three specific *Triticum turgidum* wheat genotypes that were selected in our previous investigation for their potential low toxic/immunogenic activity for celiac disease (CD) patients. The flours were subjected to sourdough fermentation with a mixture of selected *Lactobacillus* strains, and in presence of fungal endoproteases. The breads were characterized by R5 competitive enzyme linked immunosorbent assay in order to quantify the residual gluten, and the differential efficacy in gluten degradation was assessed. In particular, two of them were classified as gluten-free (<20 ppm) and very low-gluten content (<100 ppm) breads, respectively, whereas the third monovarietal prototype retained a gluten content that was well above the safety threshold prescribed for direct consumption by CD patients. In order to investigate such a genotype-dependent efficiency of the detoxification method applied, an advanced proteomic characterization by high-resolution tandem mass spectrometry was performed. Notably, to the best of our knowledge, this is the first proteomic investigation which benefitted, for protein identification, from the full sequencing of the *Triticum turgidum* ssp. *durum* genome. The differences of the proteins’ primary structures affecting their susceptibility to hydrolysis were investigated. As a confirmation of the previous immunoassay-based results, two out of the three breads made with the processed flours presented an exhaustive degradation of the epitopic sequences that are relevant for CD immune stimulatory activity. The list of the detected epitopes was analyzed and critically discussed in light of their susceptibility to the detoxification strategy applied. Finally, in-vitro experiments of human gastroduodenal digestion were carried out in order to assess, in-silico, the toxicity risk of the prototype breads under investigation for direct consumption by CD patients. This approach allowed us to confirm the total degradation of the epitopic sequences upon gastro-duodenal digestion.

## 1. Introduction

The wheat varietal selection undertaken by breeders in the last decades was tailored mainly to improve its technological and productivity-related traits; however, the latter resulted in a considerable impoverishment of the genetic diversity of wheat-based products available on the market. A recent debate supported the idea that such phenotype-based selection might have led to a greater immunogenicity of modern varieties, causing the increasing prevalence of celiac disease (CD) and other gluten-related disorders [1]. In line with this perspective, researchers focused on the natural diversity in the proteomic profile of cultivated and non-cultivated wheat genotypes, disclosing the correlations with their differential toxic potentials. Different analytical approaches and genotypes were investigated by independent working groups, all confirming similar key points [2,3,4,5,6,7,8]. First, it was assessed that there is a great variability in the immunogenic level of wheat genotypes and, although none of them can be considered safe for direct consumption by CD patients, there is an undeniable potential to select lines with lower toxicity for newly-tailored breeding programs [9,10,11]. Interestingly, such variability was investigated in old, landraces, and modern genotypes, reporting no correlation with the year of release, meaning that the past breeding programs did not cause an increase for immunostimulatory epitopes, as was originally speculated [1]. Indeed, the genetic improvement of wheat by breeders was mostly focused on the glutenin fraction, which is the main factor that is responsible for the dough’s strength and baking characteristics [12,13].

Consequently, the comprehensive proteomic characterization of wheat genotypes played a key role in providing new insight into gluten protein expression, not only supporting the drive to scout genotypes combining lower toxicity with satisfactory technological properties, but also posing convenient bases for the development of new detoxification strategies [10,14]. All of these efforts chase the long-term common objective to improve the dietary habits of people affected by CD.

The current detoxification technologies mainly rely on enzymatic-based protein hydrolysis treatment [15] or modification and sourdough-based fermentation [16,17]. Enzymes obtained from various sources (either fungi or bacteria) have been used to modify the immunogenic fraction of gluten proteins [17,18,19]. In particular, endopeptidases exhibit post-proline and/or post glutamine cleavage activity and, as such, can specifically degrade the epitopic sequences and minimize the CD-induced immunoreactivity of gluten proteins [20]. Microbial transglutaminases, which are typically used as texturizing agents in food products, have been used for gluten detoxification by the transamidation of lysine residues, which in turn reduces the binding ability of human leucocyte antigen (HLA) DQ2/8 [18]. The sourdough technology provides the fermentation of the wheat flour with naturally occurring lactic acid bacteria and yeasts. Previous studies have shown that specific lactobacillus strains can produce peptidases that are able to proteolytically cleave the gliadin fraction of wheat gluten [21,22,23,24]; however, the glutenin fraction was proved to be more resistant to microbial proteolysis [23,24].

Recently, we presented the detailed characterization of a *Triticum turgidum* wheat collection through a multidisciplinary approach [3], and we deepened the knowledge about the proteomic profile of some of the genotypes that appeared particularly promising for their gluten composition [25]. These latter were assessed in order to encrypt a reduced number of toxic/immunogenic epitopes for CD, whilst still providing the satisfactory rheological properties required for their perspective usability in bread or pasta.

In this investigation, three selected materials were used for the preparation of prototype breads from processed flours produced by the combination of sourdough fermentation (selected *Lactobacillus* strains) and enzymatic proteolysis by fungal endoproteases. The gluten content of all three genotypes was assessed by R5-competitive enzyme linked immunosorbent assay (ELISA), and a variable hydrolysis degree was accomplished for each flour. In order to increase the understanding of this experimental evidence, we carried out a detailed proteomic characterization by high resolution mass spectrometry (HR-MS). In particular, the total proteins were extracted by a strongly denaturing and reducing buffer solution that was previously optimized [26], and the discovery HR-MS analysis was carried out on both the high and low molecular weight fractions in order to disclose the amino acid sequence of the hydrolyzed and resistant peptides. Finally, the occurrence of intact CD epitopes was investigated in-silico by querying on-line databases containing all of the known CD epitopes [27]. Interestingly, for the first time, a proteomic characterization benefited from the full sequencing of the *Triticum turgidum* ssp. *durum* genome [28], providing new insights and discussions.

## 2. Materials and Methods

### 2.1. Plant Materials and Proteolytic Mixture

Three wheat genotypes were selected for the present investigation: (1) Colosseo (*Triticum turgidum* ssp. *durum*), (2) Neolatino (*Triticum turgidum* ssp. *durum*), and (3) PI 56263 (*Triticum turgidum* ssp. *turgidum*). The wheat genotypes were grown in the experimental field “A. Martucci” of the Department of Soil, Plant and Food Sciences at Valenzano (Bari, Italy) in 2017, in a randomized complete block design. The full details of the agronomic practices are described elsewhere [3].

Three lactic acid bacteria (LAB) strains belonging to the Culture Collection of the Department of Soil, Plant and Food Science were selected for the present investigation, according to their specific proteolytic activity: *Lactobacillus sanfranciscensis* GF1, *Lactobacillus plantarum* GF2, and *Lactobacillus casei* GF3. The LAB strains were cultivated for 24 h at 30 °C on MRS (de Man, Rogosa & Sharpe broth) in addition to maltose and yeast, both at 5 g/L. Commercial proteases from *Aspergillus oryzae* (500,000 hemoglobin units on the tyrosine basis/g; enzyme 1 [E1]) and *Aspergillus niger* (3000 spectrophotometric acid protease units/g; enzyme 2 [E2]), which are routinely used for bakery applications, were supplied by BIO-CAT Inc. (Troy, VA). The fungal protease Veron PS (25 g/100 kg of flour) and the protease Veron HPP (from Bacillus subtilis) (10 g/100 kg of flour) were supplied by AB Enzymes.

### 2.2. Prototype-Breads Preparation

Sourdoughs were produced from each *T. turgidum* genotype by mixing 30% (*w*/*w*) flour with 70% (*w*/*w*) tap water, this latter containing a suspension of the pooled LAB strains (*L. sanfranciscensis* GF1, *L. plantarum* GF2, *L. casei* GF3) at the density of 9 log colony forming unit/g (CFU/g). Before the sourdough fermentation, a mixture of the commercial enzymatic preparations was added. In particular, E1 and E2 were added at 200 ppm, Veron PS was added at 25 g/100 kg of flour, and Veron HPP was added at 10 g/100 kg of flour. The doughs were incubated for 48 h at 37 °C, with stirring conditions of ca. 200 rpm.

Gluten-free breads (dough yield (DY) = dough weight × 100/flour weight, of 200) were prepared using rice and corn flours (ratio 1:1 of dry matter). The sourdough was added into the final recipe of the bread (30% of the total amount of dough). Baker’s yeast was added at the percentage of 2% *w*/*w*, corresponding to a final cell density of about 9 log CFU/g in all of the breads. The doughs were mixed at 60 g for 5 min with an IM 5–8 high-speed mixer (Mecnosud, Flumeri, Italy), and the fermentation was carried out at 30 °C for 1.5 h. All of the breads were baked at 220 °C for 30 min (Combo 3, Zucchelli, Verona, Italy). The resulting breads were coded as HYD-1 (hydrolysed Colosseo flour), HYD-2 (hydrolysed Neolatino flour), and HYD-3 (hydrolysed PI 56263 flour). The control breads were prepared using untreated flours (not subjected to sourdough fermentation and without protease addition) in the same ratio and according to the same production protocol. The resulting samples were coded as CTRL-1 (Colosseo flour), CTRL-2 (Neolatino flour), and CTRL-3 (PI 56263 flour).

After baking, the breads were manually crumbled, collected in a flat box and left at 37 °C overnight for dryness. Afterwards, the crumbles were ground with a laboratory blender (Sterilmixer 12, VWR International PBI, Milano, Italy) for 30 s at 16,000 rpm (CHECK SPEED 10). The minces were carefully mixed in a plastic bag for 5 min for homogeneity, and then manually sieved with a 1 mm mesh, aliquoted, sealed under vacuum, and stored at −20 °C until their use. Three independent batches of sourdoughs and breads were produced and analysed.

### 2.3. Gluten Quantification by Immune-Enzymatic Assay (R5 Competitive ELISA)

The analysis of the gluten was carried out using the RIDASCREEN^®^ Gliadin competitive (Art. No. R7021, R-Biopharm) kit, according to the producer’s instructions. In detail, two protein extracts were prepared for each prototype bread, and each extract was assayed on two different wells of the microplate. For the control samples (CTRL-1, CTRL-2, and CTRL-3) and the processed sample (HYD-2), an additional dilution factor of 1:200 in 60% ethanol was applied in order to allow proper gluten quantification within the validated dynamic range.

### 2.4. Proteomic Characterization by High Resolution Tandem Mass Spectrometry (HR-MS/MS) Analysis

#### 2.4.1. Sample Preparation Protocol

Two hundred milligrams of the minces from all of the prototype breads were extracted with 5 mL previously-optimized sample buffer, under strong denaturant and reducing conditions (100 mM Tris HCl pH 8.5, 8 M urea and 2% *v*/*v* dithiothreitol) [26]. After the buffer’s addition, the mixture was shaken with a vortex for 2 min and subjected to a probe-based ultrasound-assisted extraction, as has previously been described [29]. Afterwards the samples were centrifuged for 10 min at 3500 *g* and the supernatants were collected.

The total protein amount of the supernatant was quantified using a commercial kit for colorimetric assays (Quick Start^TM^ Bradford protein assay, Bio-rad Laboratories).

Aliquots of such extracts (0.5 mL) were loaded on 3 kDa cut-off membranes for ultra centrifugal filtration (Amicon^®^, 3 kDa ultra centrifugal filters, Merck), which were properly activated with MilliQ water according to the instructions of the provider. The permeate fraction containing the low molecular weight compounds (<3 kDa) was labelled and referred to as the LMW fraction of the protein extract.

In addition, a 30 µL aliquot of the extract was diluted 1:10 with chymotrypsin optimized digestion buffer (Tris HCl 100 mM, pH 8.0 added with 10 mM of CaCl_2_), for a final volume of 300 µL. Such samples were thermally denaturated (15 min incubation at 95 °C under shaking, 500 rpm), chemically reduced (added 30 µL of dithiotreitol 50 mM, 30 min incubation at 60 °C under shaking 500 rpm), and alkylated (added 60 µL of iodacetamide 100 mM, 30 min incubation at RT, in the dark). The enzymatic digestion was then started by the addition of 6 µL chymotrypsin solution, 0.5 µg/µL in 1 mM HCl (minimum enzyme/protein ratio of 1:20 for each sample). The digestion was left overnight at 37 °C under shaking (500 rpm) and stopped after 15 h by acidification with 5 µL HCl 6 M. The digests were centrifuged at 13,000 rpm for 10 min. The resulting peptide pool was referred to as the high molecular weight (HMW) digests.

#### 2.4.2. Discovery HR-MS/MS Analysis and Protein/Peptides Identification

Micro-HPLC-MS/MS analyses were performed on an Ultimate 3000 UHPLC system coupled to a hybrid quadrupole-Orbitrap^TM^ mass spectrometer Q-Exactive Plus (Thermo Fisher Scientific, San Josè, CA, USA). The chromatographic separation was accomplished with an Acclaim PepMap100, C18 column, (3 μm, 100 Å, 1 × 150 mm). The untargeted HR-MS/MS analyses were performed using the Full-MS/dd-MS2 analysis mode; all of the instrumental details are described elsewhere [3,25]. The raw data were processed by Proteome Discoverer v.2.1 sp1 (Thermo Fisher Scientific) for peptide/protein identification. The Sequest HT searching algorithm was applied against a customized database including all of the entries related to *Triticum* taxonomy (https://www.uniprot.org/, accessed on 27 February 2020, total of 335,217 accessions including the *Triticum turgidum* ssp. *durum* reference proteome UP000324705). The processing workflow was set as follows: non-specific cleavage; mass tolerance on the precursor and fragment ions 10 ppm and 0.02 Da, respectively; precursor mass 300–6000 Da; minimum peak count 3; dynamic modifications: methionine-oxidation, glutamine/asparagine-deamidation, N-terminal glutamine cyclization to pyroglutamate, N-terminal protein acetylation; and static modifications: cysteine-carbamidomethylation (only for the HMW-fraction). The peptide list obtained as the software output was filtered for the best results reliability according to the following criteria: at least 2 peptide-spectrum matches (PSMs) for each sequence, and unambiguous PSM only.

### 2.5. In Vitro-Simulated Human Gastroduodenal Digestion Experiments

The minces of the breads obtained from the processed flours Colosseo (HYD-1) and PI 56263 (HYD-3) were subjected to additional experiments of in-vitro simulated human gastroduodenal (GD) digestion. The standardized static model proposed by Minekus et al. in 2014 [30] was applied to 1 g mince for each sample. After the GD digestion, the samples were purified by solid phase extraction (SPE) according to protocols reported elsewhere [3,25], with few modifications. In particular, the SPE was carried out on C18 disposable cartridges, loading 1 mL sample. After elution with 90% methanol, the samples were dried and resuspended in 100 µL of 90:10 water/acetonitrile + 0.1% formic acid to achieve a pre-concentration factor of 10 times.

The samples were analysed in the manner already described in Section 2.4.2, with a few modifications related to the software-based data processing. The GD digestion was carried out with a complex enzyme mixture; as such, no specificity was expected for the proteolytic cleavage, and the ‘no enzyme’ option was set for the data analysis. Moreover, the carbamidomethylation of the cysteine residues was excluded because the reduction/alkylation step was not included in this protocol.

## 3. Results and Discussion

### 3.1. Prototype Bread Sample Preparation

Maize, rice, sorghum, and pseudo-cereal flours, as well as their corresponding fractionated starches, are used as the main substitutes of wheat in celiac product formulations [31,32]. Among these, rice flour is the most commonly used in gluten-free (GF) bread formulations due to the fact that it is widely available and inexpensive, and is characterized by an appreciated sensory profile. It is white in colour, bland in taste, easily digested, and hypoallergenic. Despite these advantages, rice flour presents technological limitations in bread-making due to the poor functional properties of its proteins, as is also observed for all of the other GF cereal flours [33,34].

Besides the unavoidable structural and sensory problems related to the use of GF flour instead of wheat, the nutritional features of GF products are also widely debated [35]. Indeed, it was reported that GF commercial products are often characterized by very low dietary fiber content and excess calories [36,37]. Since the scientific community has correlated the unbalanced GF diet to the increasing occurrence of chronic degenerative pathologies, the necessity to improve the nutritional value of the GF products has already been highlighted [37,38]. From this point of view, sourdough fermentation was reported as an effective tool for the improvement of the sensory, technological, and especially nutritional and functional properties of GF baked goods [39]. Different research groups [39,40,41,42,43] have demonstrated that a biotechnological protocol including the use of selected sourdough lactobacilli can lead to a complete hydrolysis of gluten during the long-time fermentation of wheat flour [39]. Long-term in vivo trials have confirmed that experimental baked goods that were made with detoxified fermented flours were completely safe [41,42], thus leading to the industrialization of the process [44].

Based on the above-mentioned knowledge, a biotechnological process based on the use of three selected LAB and commercial proteases was used in this work in order to detoxify the gluten form the flours obtained from three different wheat genotypes. *L. sanfranciscensis* GF1, *L. plantarum* GF2, and *L. casei* GF3, previously isolated from wheat sourdoughs, were selected based on their protease and peptidase activities (data not shown), and were used as a mixed starter. It is well known that LAB possess a very complex peptidase system [45], although this is not a unique strain that may possess the entire pattern of peptidases needed for the hydrolysation of all of the peptides in which Pro residue are present, such as in gluten sequences. The role of the fungal proteases is retained essentially in the primary proteolysis, by releasing various sizes of polypeptides, which are thus available for the bacterial degradation.

As such, sourdoughs were used to make the GF prototype bread including rice and corn flours as the main ingredients. As we were aiming for a biochemical investigation, the prototype formulation did not include the structuring or flavouring agents commonly required to obtain products that are designed for commercial use or consumption [36].

### 3.2. Gluten Quantitation and Detoxification Efficiency

The quantitation of gluten in fermented/hydrolysed foods poses analytical challenges in method development and validation because the peptide patterns deriving from proteolysis can dramatically differ according to the fermentation/hydrolysis processes applied; theoretically, the relevant calibrants required for quantitative purposes should change accordingly. In addition, the regulatory threshold of 20 ppm was based on studies examining the immunopathogenicity of intact gluten [46,47]; whether the immunoreactivity/toxicity potential is the same for gluten peptides produced during fermentation is unknown. The protein/peptide profiles generated during the fermentation of different foods is dependent on numerous parameters, such as ingredients, time, temperature, and selected microrganisms and/or enzymes; therefore, it is an unrealistic goal to generalize the profile of different fermented/hydrolysed foods.

Until now, ELISA kits have routinely been used for the detection and quantitation of gluten in food and, in particular, the competitive assays that recognize a single epitope represent the preferential choice for the detection of immunoreactive peptides in hydrolysed foods. Competitive assays based on R5 (Ridascreen^®^ Gliadin Competitive by R-Biopharm) and G12 (GlutenTox^®^ Competitive by Biomedal Diagnostics) monoclonal antibodies are commercially available. The R5 competitive ELISA kit grounds its specificity on the R5 monoclonal antibody, which was specifically raised against the peptide sequences QQPFP, QQQFP, LQPFP, QLPFP, and includes pepsin/trypsin enzimatic digested prolamins from wheat, rye, and barley as calibrants. It features the first action approval by the association of official analytical chemists (AOAC) for the official method of analysis (OMA 2015.05) and as such, it represents the best choice currently available on the market for hydrolysed gluten quantification, and for a preliminary estimation of the efficacy in gluten detoxification strategies [48].

All of the prototypes prepared in this investigation were subjected to R5 immunoassay for residual gluten quantification. Gliadin fractions of both the hydrolysed and control samples were extracted with 60% ethanol, according to the kit’s instructions. The set dynamic range for the assay was between 10 and 270 ng/mL, corresponding to 10–270 ppm of the gluten in the food matrix. Based on this, the three CTRL samples expected to contain a gluten concentration above the kit’s upper limit were subjected to an additional dilution (1:200) of the protein extract before performing the assay, in order to allow a proper quantification within the validity range of the calibration curve. Notably, all of the tested samples were properly quantified except for the HYD-2 samples, which generated an out of range result ([gluten] > 270 ppm). The assay was repeated for this sample by applying an increasing dilution factor of the gliadin extract in order to reach the proper levels for the gluten quantification. The averaged results are reported in Table 1.

By comparing the CTRL and HYD samples for each wheat genotype, a reduced amount of gluten was assessed to be present in all of the three prototype breads from the hydrolysed flours; the reduction was directly ascribed to the proteolytic activity of the enzymatic/microbial mixture designed for the current investigation. However, the fermentation process—applied under comparable conditions to the three selected wheat flours—provided a variable efficacy in gluten degradation depending on the specific genotype. In particular, the sample prepared by the sourdough fermentation of Colosseo flour (HYD-1) resulted in a final prototype bread which can be labelled as GF, due to residual gluten content below the 20 ppm threshold limit (11.3 ± 1.3 ppm). The prototype bread HYD-3, prepared with PI 56263 flour, presented a residual gluten concentration of 36 ± 7 ppm, which is referred to as ‘very low gluten content’ (20 ppm < [gluten] < 100 ppm). In both cases, the gluten degradation efficiency was assessed to be very high (≥99.5%), and the final prototype breads could potentially be included in the diet of CD patients. On the contrary, the HYD-2 sample prepared using Neolatino flour maintained a high level of residual gluten (7600 ± 700 ppm), and was thus not acceptable for direct consumption by CD patients. Notably, in this investigation, it was proven for the first time that the efficiency of gluten detoxification strategies are strictly related to the specific protein profile of the wheat flour. Interestingly, the genotype-depending efficiency reported here poses specific challenges to food technologists because it constrains the validity of all of the previous investigations dealing with gluten hydrolysis by the enzymatic treatment and/or sourdough fermentation of the specific flours on which they were developed and validated. In order to increase the understanding of this experimental evidence, we carried out a detailed proteomic characterization using high resolution tandem mass spectrometry (HR-MS/MS).

### 3.3. Proteome Profiling and Resistant Epitope Matching

**Figure 1:**
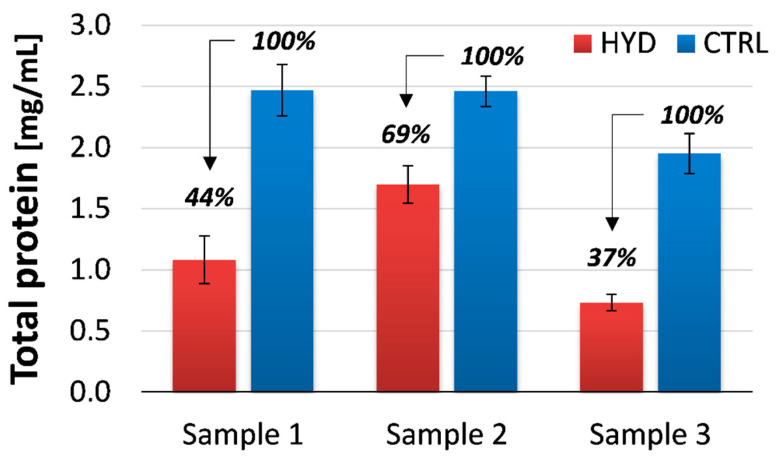
Protein quantification of the model breads by Bradford colorimetric assay carried out on the total protein extracts.

For the proteomic characterization of prototype samples, a comprehensive protein extraction was carried out under previously optimized conditions [26]. A strong denaturing and reducing buffered solution was prepared and applied to all of the bread minces (CTRL and HYD). The total protein extracts were quantified using a commercial kit with two analytical replicates and two technical replicates. Figure 1 summarizes the averaged results. Notably, all of the extracts derived from the processed flours (HYD-1, HYD-2, HYD-3) presented a protein concentration lower than the relevant control samples (CTRL-1, CTRL-2, CTRL-3, respectively). The rationale of this experimental evidence was found in the working principle of the colorimetric assay. Indeed, as a Coomassie dye-based assay, the development of colour is associated to the instauration of Van der Waals forces, and hydrophobic interactions between the dye and specific side chains of the proteins. Peptides or oligopeptides with low molecular weights (<3 kDa) cannot provide such an interaction and do not produce colour in reaction to Coomassie dye reagents. Therefore, the results reported in Figure 1 presented an indirect confirmation of the protein hydrolysis occurring in all of the hydrolysed flours at different degrees.

In order to characterize the low-molecular weight (LMW) fraction resulting from flour hydrolysis, the ultrafiltration of the total protein extracts on cut-off membranes was applied, with a size limit of 3 kDa. The permeate fraction (LMW) was directly analysed by untargeted HR-MS/MS for the sequence identification. For comparison, we applied the same protocol and analysis to both the hydrolysed and control breads, notwithstanding the absence in the latter of the fermentation/hydrolysis step.

The HR-MS/MS analysis was carried out in data dependent acquisition mode, and the fragmentation spectra were processed via commercial software for sequence identification against a customized database containing all of the protein accessions currently assigned to the *Triticum* taxonomy. Notably, such a database was significantly extended compared to our previous investigation [25], because it was populated with the whole proteome of *Triticum turgidum subsp. durum* (taxonomy ID 4567), which was made publicly available after the full sequencing of its genome [28]. Therefore, we expected, for this investigation, a wide coverage and good reliability in the peptide and protein sequence identification, benefitting from the *T. turgidum subsp. durum* reference proteome. Given the high complexity of the proteolysis accounted for by the simultaneous microbial and fungal activities, a non-specific cleavage was set for the database indexing. As was expected, very few peptides were detected in the LMW fraction of the three control samples, confirming that all of the sequences identified in samples HYD-1, HYD-2, and HYD-3 were directly ascribed to the detoxification strategy devised and carried out on these samples. In particular, 312, 242 and 384 peptides were detected in samples HYD-1, HYD-2, and HYD-3, respectively, as is reported in Table 2.

**Figure 2:**
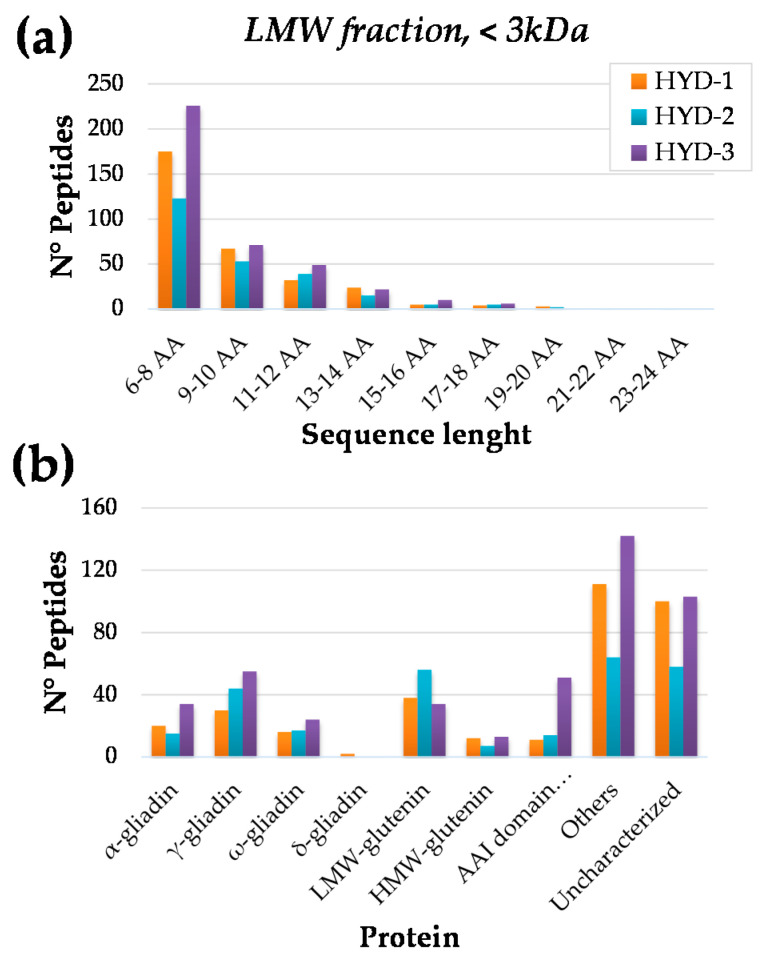
Overview of the peptides identified in the low molecular weight fraction (LMW, <3 kDa) by the HR-MS/MS analysis. Panel (**a**): peptide count according to the sequence length. Panel (**b**): peptide count according to the belonging protein (please consider that several peptides were shared among different accessions).

Figure 2 presents an overview of the detected peptides according to their specific features. In particular, the size distribution was reported in Figure 2a, expressed as the number of amino acid (AA) residues. For all three samples (HYD-1, HYD-2, HYD-3), most of the identified sequences resulted in very short fragments (6–8 AA) that featuring a length below the minimum peptide-binding register (cut-off 9 AA) did not pose any toxicity risk in CD patients [27]. Notably, the previous comments about a differential hydrolysis accounted for by the microbial/enzymatic activity on the three monovarietal flours was consistently supported by such a preliminary MS investigation on the LMW fraction. Indeed, also in this experiment, the sample HYD-2 results showed that it was less affected by protein degradation than the other two samples, resulting in a lower count of identified peptides, especially as for the shortest fragments. In Figure 2b, the peptide distribution among the different proteins is displayed, with particular attention to the storage proteins that were individually counted, whereas all of the metabolic and other water-soluble proteins were listed in the general category ‘others’, and all of the accessions that were not directly ascribable to the previous categories were labelled as ‘uncharacterized’.

These latter two classes of proteins were not taken into consideration for further discussion because they were not relevant for the CD immunoreactivity perspective. Interestingly, all of the storage proteins, namely gliadins (α-, γ-, ω-, δ-type) and glutenin (low molecular weight, LMW and high molecular weight, HMW) were affected, to a certain extent, by the microbial/enzymatic degradation. Of particular interest was the ability to hydrolyze α- and γ- gliadins, especially in the sample HYD-3, as they are mainly responsible for the toxicity level of durum wheat flours towards CD patients. In addition, the protein accessions referred to as ‘AAI domain-containing proteins’ were counted as an independent category because they featured partial sequence homology with γ-gliadin, α-gliadin, and LMW-glutenin accessions, and thus deserved attention for the toxicity risk evaluation.

Such an evaluation was accounted for by an in-silico assessment of the sequence identity with known T-cell epitopes [27] by means of the CELIAC Database v.2 and the relevant tool for protein risk assessment (http://www.allergenonline.org/celiacfasta.shtml). All of the peptides detected in the LMW fractions were searched for an exact match with the immunostimulatory sequences, and the positive matches were counted in Table 2. Notably, only a few sequences from γ-gliadins (featuring partial sequence homology with ω-gliadins) included intact epitopes, and they were detected only in sample HYD-2 and HYD-3, whereas no intact epitope was found in sample HYD-1.

In order to investigate further the protein degradation accounted for by the sourdough fermentation with the mixture of L. strains and fungal proteases, the high molecular weight (HMW) fraction of the protein extracts was also characterized by untargeted HR-MS/MS analysis. A typical workflow for a bottom-up proteomic approach, with chymotrypsin as specific enzyme, was applied to both the HYD and CTRL samples, keeping the latter as the internal reference to trace back to the susceptible and resistant sequences. Hundreds of sequences ascribed to *Triticum* taxonomies were identified in all of the samples, especially in the protein extract from the CTRL prototype breads (see Table 2 for the specific counting). Again, the difference in the number of detected peptides can likely be ascribed to the protein degradation occurring during the flours’ fermentation, as this step was the only difference in the production of the HYD and CTRL samples. All of the identified peptides were screened for an exact match with known T-cell epitopes, as was previously described, after filtering out all of the sequences lower than 9 AA in length. As was expected, the three control samples encoded for several tens of intact T-cell epitopes, which were differently distributed among the main storage proteins (see Table 2). Interestingly, protein accessions generally described as AAI domain-containing proteins (A0A446IHC0; A0A446IHA8, B6UKQ6, etc.) actually coded for full length epitopes; indeed, 8, 11, and 21 hazard peptides were detected in samples CTRL-1, CTRL-2, and CTRL-3, respectively (see Table 2). Notably, no intact epitope was detected in the HMW-fraction of sample HYD-1, and only one hazard peptide was detected in the HMW-fraction of HYD-3, proving that the detoxification strategy was very efficient for these two flours, degrading almost completely the epitopes coded by each genotype down to concentration levels below the sensitivity of this analytical method (see Table 2). On the contrary, a very different result was obtained for the HMW-fraction of the HYD-2 sample, in which most of the epitopes detected in the CTRL-2 sample were resistant to the proteolytic activity of the selected L. strains and peptidases involved in the fermentation process.

The untargeted HR-MS/MS analysis that was carried out proved unequivocally that—notwithstanding that the fermentation was equally applied to all three flours—its efficacy in gluten detoxification was dramatically different according to the specific genotype. Since all three genotypes were systematically characterized in our previous investigation [25], and were all promising in terms of reduced gluten content and potential lower toxicity, such differential behavior upon subjection to the fermentation can only find explanation in punctual differences of the protein primary structure that affects their susceptibility to hydrolysis by microbial/fungal proteases. According to this speculation, we carefully evaluated the list of peptides containing intact epitopes and grouped them based on the specific epitope that was coded. In addition, we also disclosed, whenever available, the relevant restricted 9 AA core epitope according to the current nomenclature proposed by Sollid et al. 2020 [49], in order to streamline the reading and understanding of the results. Indeed, most of the detected epitopes, which referred to different identification numbers in the CELIAC Database actually shared the same core 9 AA epitope, and thus likely presented similar binding efficiency to HLA-DQ antigens. Finally, thanks to the parallel analysis carried out on the control samples of each genotype, we furtherly deepened the data analysis by classifying the detected epitopes as ‘resistant’ or ‘susceptible’ to the fermentation process applied. Namely, the resistant epitopes were the ones identified in either the LMW or HMW fraction of the processed breads (HYD), whereas the susceptible epitopes were the sequences detected in any of the CTRL samples, but that were missing in the relevant HYD sample, thus suggesting its likely hydrolysis by the fermentation. Table 3 and Table 4 present the results of such a data analysis, reporting the susceptible and resistant epitopes, respectively. The epitopes were listed according to the identification numbers assigned to them by the CD database (http://www.allergenonline.org/celiachome.shtml) and some further information about toxicity and the HLA-DQ antigen, as well as the number of peptides per sample encrypting the specific epitope. As was already mentioned, the possibility to identify the peptide sequences by searching against the reference proteome of durum wheat provided an undeniable advance for the current investigation. Browsing the list of the identified epitopes (see Table 3 and Table 4), several sequences encrypting the 9AA-cores DQ2.5-glia-α1b, DQ2.5-glia-α2, DQ8-glia-α1,a DQ2.5-glia-γ4b, expected to be coded only by the D genome, were detected [50]. Therefore, this proteomic investigation represents—to the best of our knowledge—the first experimental evidence that such epitopic sequences can also be expressed in tetraploid wheats.

Assessing whether the detoxification strategy provided the efficient degradation of the specific DQ2.5 and/or DQ8 epitopes boasts great relevance from the general perspective, because CD patients may express foremost either one. Indeed, approximately 95% of CD patients express HLA-DQ2.5 antigens, which are then statistically more relevant for the susceptible population, whereas the rest are usually either HLA-DQ8 positive, or, to a minor extent, express HLA antigens that contain only one of the DQ2.5-chains, e.g., DQ2.2 or DQ7.5 [49,51]. In addition, although polyclonal T-cell recognizing multiple epitopes are usually detected in CD patients, specific responses to the DQ2.5-glia-α1, DQ2.5-glia-α2 epitopes, and homologues thereof (ω-gliadins, hordeins and secalins) are dominant in DQ2.5-positive patients, and responses to the DQ8-glia-α1 epitope are most frequently found in DQ8-positive patients [27].

Notably, the current investigation proved that the devised protocol for gluten detoxification enabled the efficient degradation of the main core epitopes DQ2.5-glia-α1b, DQ2.5-glia-α2, DQ2.5-glia-α3, DQ2.5-glia-ω1, DQ2.5-glia-ω2, and DQ2.5-hor-2, which are differently expressed in the three genotypes under investigation (see Table 3); moreover, under the same fermentation conditions, the core epitopes DQ2.5-glia-γ1/DQ8.5-glia-γ1, DQ2.5-glia-γ4b, and DQ2.5-glut-L2 were completely hydrolysed. In addition, from a further analysis of the list reported in Table 3, it was found that also the DQ2 epitopes with ID numbers 182, 188, 222, 231, 236, 501, 502, 504, 611, 731, 950, 973, 1040, 1042, and 1044, and the DQ8 epitopes with ID numbers 119, 140, 502, and 611 were all completely susceptible to the detoxification applied. On the contrary, the epitopes containing the core sequences DQ2.5-glia-γ4c/DQ8-glia-γ1a (shared sequence) presented only a partial susceptibility to the hydrolysis, depending on the specific ID number (see Table 3 and Table 4). Finally, in Table 4, all of the alternative full-length epitopes that survived the fermentation process, which were still detectable in the hydrolysed sample, were reported. Most of these resistant epitopes were actually found only in the HYD-2 sample, since, as was already mentioned, no T-cell activating sequence was detected in the HYD-1 protein sample (neither in the LMW nor in the HMW fractions), and only few peptides were found in the HYD-3 sample. In particular, the epitopes containing the cores DQ2.5-glia-α1a, DQ8-glia-α1, DQ2.5-glia-γ2, DQ2.5-glia-γ3/DQ8-glia-γ1b, DQ2.5-glia-γ5, and DQ2.5-glut-L1 were proven to be resistant to the hydrolysis, together with several other epitopic sequences belonging mainly to α-gliadins, and less so to γ-gliadin, ω-gliadin, and glutenin (see Table 4 for the full list).

### 3.4. In Vitro-Simulated Human Gastroduodenal Digestion Experiments and In-Silico Evaluation of the Toxicity Risk for Celiac Disease Patients

As a final step, the two prototype breads of main interest, HYD-1 and HYD-3, which were shown to be GF and low-gluten content, respectively, by immunoassays, were subjected to in-vitro simulated human gastroduodenal (GD) digestion experiments. The aim was to evaluate the digestibility and toxicity risk for CD patients of the hydrolyzed gluten proteins in such processed samples, in experimental conditions, which simulate the human GD digestion process. The standardized static protocol applied [30] provided all of the technical details required simulating in-vitro the three main steps of the physiological process, namely the oral, gastric and duodenal phases. The protein digestion was assessed only at the end point of the whole process; after the 2 h incubation occurring in the duodenal phase, the procedure was stopped with phenylmethyl sulfonyl fluoride, and the peptide pools were purified by solid phase extraction on disposable cartridges. The resulting purified samples were characterized by untargeted HR MS/MS analysis, as described in the experimental section. The software-based identification was performed with the same database applied to the previous proteomic investigation by setting an unspecific cleavage for the peptide sequence assignments, due to the high complexity of the enzyme mixtures involved into the human GD process.

Notably, the final lists of peptide sequences assigned to *triticum*-belonging proteins was quite short; only 291 peptides for the GD digest of bread HYD-1, and 227 peptides for the GD digest of bread HYD-3. This proved that the extensive hydrolysis affected the wheat proteins after the combined effect of the detoxifying strategy and the GD digestion process. Moreover, an in-depth analysis of the data acquired showed that, in both cases, most of the peptides detected were below the 9 AA length cut-off, and thus do not pose a risk to elicit any immune-response in CD patients. In particular, only 79 peptides out of the 291 detected for the HYD-1 bread were greater than or equal to 9 AA in length. Similarly, only 71 peptides out of the 227 detected were greater than or equal to 9 AA in length for the HYD-3 bread.

These short lists of sequences were screened against the CELIAC Database, as previously described, in order to disclose the presence of epitopic sequences that were resistant to the detoxification process applied and survived the GD digestion, as well. Notably, no epitope was detected in the GD digests of both the HYD-1 and HYD-3 breads. This experimental evidence confirmed that the detoxification strategy applied to these two prototype samples was successful in hydrolyzing the toxic/immunogenic sequences expressed in the relevant monovarietal flours down to concentration levels thar become not detectable in the in-vitro simulated human GD digests.

## 4. Conclusions

In this investigation, we reported the production of prototype GF breads from processed flours of specific *Triticum turgidum* wheat genotypes, which were subjected to sourdough fermentation with a mixture of selected *Lactobacillus* strains and fungal endoproteases. The immunoassay-based characterization suggested a differential efficiency in the gluten degradation according to the specific monovarietal flour, which was investigated in-depth by HR mass spectrometry and in-silico epitope mapping. The in-vitro simulated human GD experiments also proved the absence of toxic/immunogenic epitopes that are relevant for CD patients in the prototype breads produced, confirming the relevance of this investigation for the improvement of the dietary habits of vulnerable individuals. Notably, the advanced proteomic analysis provided new insight for the development of detoxification strategies assessing a genotype-depending efficiency of the proteolytic activity strictly related to the punctual differences of the primary protein structure. Taking advantage of the full sequencing of the durum wheat genome, a detailed list of the susceptible and resistant epitopic sequences was achieved in the current investigation, suggesting the need to constrain the validity of any detoxification strategy to the specific flours on which they are developed.

## Figures and Tables

**Table 1:** Overview of R5-competitive enzyme linked immunoassay (ELISA) analysis carried out on the prototype breads (*n* = 2 independent extracts/bread and *n* = 2 assay replicates/extract).

Sample Code	Gluten [mg/kg]	Relative Standard Deviation %	Degradation Efficiency
HYD-1 *	11.3 ± 1.3	11%	99.5%
CTRL-1 *	2490 ± 80	3%	
HYD-2 *	7600 ± 700	9%	22.0%
CTRL-2 *	9700 ± 1100	11%	
HYD-3 *	36 ± 7	19%	99.6%
CTRL-3 *	9800 ±1100	11%	

* HYD: processed bread; CTRL control bread; sample 1: bread produced with Colosseo flour; sample 2 bread produced with Neolatino flour; sample 3: bread produced with PI 56263 flour.

**Table 2:** Summary of the peptides identified by the discovery HR-MS/MS analysis of the model breads prepared with monovarietal durum wheat flour and subjected (HYD) or not (CTRL) to sourdough and enzymatic fermentation.

Sample Type	Peptides Count		HYD-1	CTRL-1	HYD-2	CTRL-2	HYD-3	CTRL-3
Low molecular weight (LMW) fraction, < 3 kDa	total identified		312	4	242	4	384	7
	hazard peptides with intact celiac disease (CD) epitope		-	-	7	-	6	-
	Protein distribution of hazard peptides *	γ-gliadin	-	-	7	-	6	-
		ω-gliadin	-	-	2	-	1	-
High molecular weight (HMW) fraction, chymotrypsin digest	total identified		614	1394	1097	1671	663	1599
	hazard peptides with intact CD epitope		-	69	46	83	1	92
	Protein distribution of hazard peptides *	α-gliadin	-	14	11	19	-	23
		γ-gliadin	-	22	20	34	-	32
		ω-gliadin	-	14	7	14	1	24
		LMW-glutenin	-	25	11	21	-	12
		HMW-glutenin	-	1	1	1	-	1
		AAI domain containing	-	8	9	11	-	21

* Several sequences were shared among the different accessions.

**Table 3:** List of the CD epitopes identified in the prototype breads (CTRL) that were susceptible to the proteolysis carried out by the selected strains of L. strains and fungal enzymes; none of them were detected in the HYD samples. The sequence reported in bold and underlined represents the 9AA core T-cell–activating epitope, according to Sollid et al. 2020 [49]. The human leucocyte antigen (HLA) was reported whenever specified.

Epitopes Search *						N° of Hazard Peptides/Sample **		
ID	Type	Toxicity ***	HLA-DQ	Sequence	Core T-Cell Epitope	CTRL-1	CTRL-2	CTRL-3
55	α-gliadin	I	DQ2	**PQPQLPYPQPQLPY**	DQ2.5-glia-α1b, DQ2.5-glia-α2	0	1	1
64	α-gliadin	I	DQ2	**PQPQLPYPQ**PQL	DQ2.5-glia-α2	0	1	1
66	α-gliadin	I	DQ2	**PQPQLPYPQ**PQ	DQ2.5-glia-α2	0	1	1
68	α-2 gliadin	I	DQ2.5	**PQPQLPYPQ**	DQ2.5-glia-α2	0	1	1
72	α-gliadin	I	DQ2	PQL**PYPQPQLPY**	DQ2.5-glia-α1b	0	1	1
84	α-3 gliadin	I	DQ2.5	**PYPQPQLPY**	DQ2.5-glia-α1b	0	1	1
93	α-20 gliadin	I	DQ2.5	**FRPQQPYPQ**	DQ2.5-glia-α3	1	1	1
119	α-gliadin	I	DQ8	GSFQPSQQNPQAQGS		0	1	0
140	α-gliadin	I	DQ8	QLIPCMDVVL		1	0	1
182	α-gliadin	I	DQ2	LQPFPQPQPFLPQLPYPQPQ		1	1	1
188	α-gliadin	I	DQ2	FPGQQQQFPPQQPYPQPQPF		1	0	1
221	ω-II gliadin	I	DQ2	**PQPQQPFPW**	DQ2.5-glia-ω2	0	1	0
222	ω-gliadin	I	DQ2	PFPWQPQQPFPQ		1	1	0
226	ω-gliadin	I	DQ2	**QQPQQPFPQ**PQLPFPQQSEQ	DQ2.5-glia-γ4c/DQ8-glia-γ1a	1	1	0
231	ω-gliadin	I	DQ2	PFPQPQQPIPV		1	1	1
236	ω-gliadin	I	DQ2	PFP**LQPQQPFPQ**	DQ2.5-glia- γ4e	0	0	1
463	γ-gliadin	I	DQ8 (DQ2/8)	QQPYP**QQPQQPFPQ**	DQ2.5-glia-γ4c/DQ8-glia-γ1a	0	1	1
501	γ1-gliadin	I	DQ2	PQQPFPQPQQTFPQQPQLPF		0	0	1
502	γ1-gliadin	I	DQ2, DQ8	PFPQPQQTFPQQPQLPFPQQ		0	0	1
504	γ1-gliadin	I	DQ2	PQQTFPQQPQLP		0	0	1
523	γ1-gliadin	I	DQ2	QQ**PQQSFPQQQ**	DQ2.5-glia-γ1/DQ8.5-glia-γ1/DQ8-glia-γ2	1	3	1
524	γ1-gliadin	I	DQ2	Q**PQQSFPQQQ**	DQ2.5-glia-γ1/DQ8.5-glia-γ1/DQ8-glia-γ2	2	3	1
530	γ-gliadin	I	DQ8 (DQ2/8)	QFPQT**QQPQQPFPQ**	DQ2.5-glia-γ4c/DQ8-glia-γ1a	0	0	1
536	γ-gliadin	I	DQ2, DQ8	QQPQLPFP**QQPQQPFPQ**PQQ	DQ2.5-glia-γ4c/DQ8-glia-γ1a	1	0	0
537	γ-gliadin	I	DQ8 (DQ2/8)	QLPFP**QQPQQPFPQ**	DQ2.5-glia-γ4c/DQ8-glia-γ1a	1	2	0
573	γ-gliadin	I	DQ2	F**PQPQQQFPQ**PQ	DQ2.5-glia-γ4b	0	0	1
577	γ-gliadin	I	DQ2.5	**PQPQQQFPQ**	DQ2.5-glia-γ4b	0	0	1
583	γ-1 gliadin	I	DQ2.5/DQ8	**PQQSFPQQQ**	DQ2.5-glia-γ1/DQ8.5-glia-γ1/DQ8-glia-γ2	2	4	2
611	γ-gliadin	I	DQ2 (DQ2.5)	PHQPQQQVPQPQQPQQPF		0	1	0
617	γ-gliadin	I	DQ8 (DQ2/8)	PFPQL**QQPQQPFPQ**	DQ2.5-glia-γ4c/DQ8-glia-γ1a	1	1	0
640	γ-gliadin	I	DQ2	Q**PQQSFPQQQ**RP	DQ2.5-glia-γ1/DQ8.5-glia-γ1/DQ8-glia-γ2	1	0	0
721	LMW glutenin	I	DQ2	QQQQPP**FSQQQQSPF**SQQQQ	DQ2.5-glut-L2	1	1	1
729	LMW glutenin	I	DQ2	QQPP**FSQQQQSPF**SQ	DQ2.5-glut-L2	2	1	1
731	LMW glutenin	I	DQ2	QQPPFSQQQQSP		5	1	2
733	LMW glutenin	I	DQ2	QPP**FSQQQQSPF**SQ	DQ2.5-glut-L2	3	2	1
734	LMW glutenin	I	DQ2	PP**FSQQQQSPF**SQQQ	DQ2.5-glut-L2	2	1	1
736	LMW glutenin	I	DQ2	P**FSQQQQSPF**SQQQQ	DQ2.5-glut-L2	2	1	1
738	LMW glutenin	I	DQ2	P**FSQQQQSPF**	DQ2.5-glut-L2	6	2	2
747	glut-L2	I	DQ2.5	**FSQQQQSPF**	DQ2.5-glut-L2	6	2	2
835	Hordein	I	DQ2	Q**PFPQPQQPF**PL	DQ2.5-glia-ω1	1	1	1
867	hor-1	I	DQ2.5	**PFPQPQQPF**	DQ2.5-glia-ω1	1	1	4
878	Hordein	I	DQ2	Q**PFPQPQQPF**SW	DQ2.5-glia-ω1	0	0	1
886	γ-hordein	I	DQ2	QQF**PQPQQPFPQ**QP	DQ2.5-hor-2	0	0	1
890	γ-hordein	I	DQ2	QQF**PQPQQPFPQ**	DQ2.5-hor-2	0	0	1
891	hor-2	I	DQ2.5	**PQPQQPFPQ**	DQ2.5-hor-2	0	1	3
930	γ-secalin	I	DQ2	QSI**PQPQQPFPQ**	DQ2.5-hor-2	0	0	1
950	ω-Secalin	I	DQ2	QPFPQPQQPIPQ		1	1	0
973	ω-Secalin	I	DQ2	IIPQQPQQPFPL		0	1	1
1040	glia-ω 3	I	DQ2.5	PFPQPQQPI		2	2	1
1042	glia-ω 4	I	DQ2.5	PQPQQPIPV		1	1	1
1044	glia-ω 5	I	DQ2.5	**LQPQQPFPQ**	DQ2.5-glia-γ4e	4	1	4

* http://www.allergenonline.org/celiachome.shtml (Accessed on 1–3 April 2020). In the case of the glutamate residues (E) expected in-vivo by TG2-mediated deamidation, the respective sequence with unmodified glutamine (Q) residue was searched. ** The number of peptides reported in brackets refers to the analysis of the LMW-fraction, whereas all of the other counts refer to the analysis of the HMW-fraction. *** I: immunogenic; T: toxic.

**Table 4:** List of CD epitopes identified in the prototype breads from the hydrolyzed flours (HYD) that were resistant to the proteolysis carried out by the selected strains of L. strains and fungal enzymes. The sequence reported in bold and underlined represents the 9AA core T-cell activating epitope, according to Sollid et al. 2020 [49].

Epitopes Search *						N° of Hazard Peptides/Sample **		
ID	Type	Toxicity ***	HLA-DQ	Epitope Sequence	Core T-Cell Epitope	HYD-1	HYD-2	HYD-3
1	α-gliadin	T	Unknown	VPVPQLQPQNPSQQQPQEQVPL	-	0	1	0
3	α-gliadin	I	DQ2	VRVPVPQLQPQNPSQQQPQ	-	0	1	0
5	α-gliadin	I	DQ2	FPGQQQPFPPQQPYPQPQPF	-	0	1	0
7	α-gliadin	I, T	HLA-DR	PQPQPFPSQQPY	-	0	3	0
14	α-gliadin	I	DQ2	LQLQ**PFPQPQLPY**	DQ2.5-glia-α1a	0	1	0
24	α-gliadin	I	DQ2	QLQ**PFPQPQLPY**	DQ2.5-glia-α1a	0	1	0
32	γ-gliadin	I	DQ2	P**QQPFPQQPQ**Q	DQ2.5-glia-γ5	0	0	0 (1)
36	α-gliadin	I	DQ2	LQ**PFPQPQLPY**	DQ2.5-glia-α1a	0	1	0
42	α-gliadin	I	DQ2	Q**PFPQPQLPY**	DQ2.5-glia-α1a	0	1	0
53	α-9 gliadin	I	DQ2.5	**PFPQPQLPY**	DQ2.5-glia-α1a	0	1	0
95	α-gliadin	I	DQ8 (DQ2/8, DQ1/8)	QQPQQQYPSG**QGSFQPSQQ**NPQAQG	DQ8-glia-α1	0	1	0
96	α-gliadin	I	DQ8	QQPQQQYPSG**QGSFQPSQQ**NPQAQ	DQ8-glia-α1	0	1	0
100	α-gliadin	I	DQ8	QPQQQYPSG**QGSFQPSQQ**NP	DQ8-glia-α1	0	1	0
101	α-gliadin	I	DQ8 (DQ2/8, DQ1/8)	QQYPSG**QGSFQPSQQ**NPQ	DQ8-glia-α1	0	1	0
102	α-gliadin	I	DQ8	QYPSG**QGSFQPSQQ**NPQA	DQ8-glia-α1	0	1	0
104	α-gliadin	I	DQ8	YPSG**QGSFQPSQQ**NP	DQ8-glia-α1	0	1	0
105	α-gliadin	I	DQ8 (DQ2/8)	PSG**QGSFQPSQQ**NPQAQG	DQ8-glia-α1	0	1	0
106	α-gliadin	I	DQ8 (DQ2/8)	PSG**QGSFQPSQQ**	DQ8-glia-α1	0	1	0
107	α-gliadin	I	DQ8 (DQ2/8)	PSGQGSFQPSQ	-	0	1	0
108	α-gliadin	I	DQ8 (DQ2/8)	SG**QGSFQPSQQ**N	DQ8-glia-α1	0	1	0
113	α-gliadin	I	DQ8 (DQ2/8)	GQGSFQPSQ	-	0	1	0
115	α2 gliadin	I	DQ8 (DQ2/8)	**QGSFQPSQQ**	DQ8-glia-α1	0	1	0
138	α-gliadin	I	DQ2	PQQPYPQPQPQ	-	0	1	0
146	α-gliadin	I	DQ2	QVPLVQQQQFLGQQQPFPPQ	-	0	1	0
149	α-gliadin	I, T	Unknown	LGQQQPFPPQQPYPQPQPFPSQQPY	-	0	1	0
150	α-gliadin	I, T	DQ2 (α1*0501, α1*0201)	LGQQQPFPPQQPYPQPQPF	-	0	1	0
151	α-gliadin	I	DQ2 (α1*0501, α1*0201)	LGQQQPFPPQQPYPQPQ	-	0	1	0
152	α-gliadin	I, T	HLA-DR	LGQQQPFPPQQPY	-	0	2	0
185	α-gliadin	I	DQ2	QPQPFLPQLPYPQP	-	0	1	0
187	α-gliadin	I	DQ2	PQPFLPQLPYPQ	-	0	1	0
195	ω-gliadin	I	DQ2	P**QQPFPQQPQ**QP	DQ2.5-glia-γ5	0	2 (2)	0
227	ω-gliadin	I	DQ2	QPFPQPQLPFPQ		0	1	0
229	ω-gliadin	I	DQ2	PFP**QQPQQPFPQ**	DQ2.5-glia-γ4c/DQ8-glia-γ1a	0	3 (1)	0 (1)
246	ω5-gliadin/LMW glutenin	I	DQ2	QQQQIPQQPQQF	-	0	1	0
252	ω5-gliadin/LMW glutenin	I	DQ2	QIPQQPQQF	-	0	2	0
426	γ-gliadin	I	DQ2	P**QQPFPQQPQQPYPQ**QP	DQ2.5-glia-γ3/DQ8-glia-γ1b, DQ2.5-glia-γ5	0	1	0
427	γ-gliadin	I	DQ2	P**QQPFPQQPQ**QPY	DQ2.5-glia-γ5	0	1	0
432	γ-gliadin	I	DQ2	P**QQPFPQQPQ**Q	DQ2.5-glia-γ5	0	2 (2)	0
437	γ-gliadin	I	DQ2	**QQPFPQQPQQPYPQ**	DQ2.5-glia-γ3/DQ8-glia-γ1b, DQ2.5-glia-γ5	0	1	0
438	γ5 gliadin	I	DQ2.5	**QQPFPQQPQ**	DQ2.5-glia-γ5	0	4 (2)	0 (1)
441	γ-gliadin	I	DQ2	PFP**QQPQQPYPQ**QPQ	DQ2.5-glia-γ3/DQ8-glia-γ1b	0	1	0
445	γ-gliadin	I	DQ2	PFP**QQPQQPYPQ**	DQ2.5-glia-γ3/DQ8-glia-γ1b	0	1	0
446	γ-gliadin	I	DQ8, DQ2	FP**QQPQQPYPQ**QPQQ	DQ2.5-glia-γ3/DQ8-glia-γ1b	0	1	0
451	γ-gliadin	I	DQ2	FP**QQPQQPYPQ**QP	DQ2.5-glia-γ3/DQ8-glia-γ1b	0	1	0
454	γ-gliadin	I	DQ2	FP**QQPQQPYPQ**Q	DQ2.5-glia-γ3/DQ8-glia-γ1b	0	1	0
458	γ1 and γ5 gliadin	I	DQ2.5/DQ8	**QQPQQPYPQ**	DQ2.5-glia-γ3/DQ8-glia-γ1b	0	2 (1)	0 (1)
464	γ-gliadin	I	DQ2	QQPYPQQPQ	-	0	1 (1)	0 (1)
468	γ-gliadin	I	DQ2 (DQ2.2 and DQ2.5)	PYPQQPQQP	-	0	1	0
472	γ-gliadin	I	DQ2.5/DQ8	**QQPQQPFPQ**	DQ2.5-glia-γ4c/DQ8-glia-γ1a	0	8 (4)	0 (4)
479	γ1-gliadin	I	DQ2	QVDPSGQVQWPQ	-	0	3	0
503	γ1-gliadin	I	DQ2	PFPQPQQTFPQ	-	0	1	0
538	γ-gliadin	I	DQ2	PFPQQPQQPF	-	0	3 (1)	0 (1)
542	γ-gliadin	I	DQ2	FPQQPQQPF	-	0	4 (1)	0 (1)
553	γ-gliadin	I	DQ8 (DQ2/8)	PFPQT**QQPQQPFPQ**	DQ2.5-glia-γ4c/DQ8-glia-γ1a	0	1	0
555	γ-gliadin	I	DQ8 (DQ2/8)	PFPQS**QQPQQPFPQ**	DQ2.5-glia-γ4c/DQ8-glia-γ1a	0	1	0
587	γ-gliadin	I	DQ2	VQGQGI**IQPQQPAQL**	DQ2.5-glia-γ2	0	3 (1)	0
593	γ-gliadin	I	DQ2	GI**IQPQQPAQL**	DQ2.5-glia-γ2	0	4 (1)	0
595	γ-gliadin	I	DQ2	I**IQPQQPAQL**	DQ2.5-glia-γ2	0	4 (1)	0
597	γ-gliadin	I	DQ2	IIQPQQPAQ	-	0	6 (1)	0
599	γ5 gliadin	I	DQ2.5	**IQPQQPAQL**	DQ2.5-glia-γ2	0	4 (1)	0
612	γ-gliadin	I	DQ8 (DQ2/8)	**QQPFPQQPQQPFPQ**	DQ2.5-glia-γ4c/DQ8-glia-γ1a, DQ2.5-glia-γ5	0	3 (1)	0
650	γ-gliadin	I	DQ2	QPFPQLQQPQQP	-	0	1	0
659	LMW glutenin	I	DQ2	QAFPQPQQTFPH	-	0	1	0
701	γ-gliadin or LMW glutenin	I	DQ2	QQP**PFSQQQQPV**LPQ	DQ2.5-glut-L1/DQ2.2-glut-L1	0	3	0
706	Glut-L1	I	DQ2.2	**PFSQQQQPV**	DQ2.5-glut-L1/DQ2.2-glut-L1	0	7	0
720	LMW glutenin	I	DQ2	QQPPFSQQQQPPFSQ	-	0	2	0
762	LMW glutenin	I	DQ2	QQPPFSQQQQQPILL	-	0	1	0
763	LMW glutenin	I	DQ2	QPPFSQQQQQPILL	-	0	1	0
781	HMW-Glutenin	I	DQ8 (DQ2/8)	GQPGYYPTSPQQPGQ	-	0	1	0
903	Secalin	I	DQ2	PQQSFPQQP	-	0	0	1
926	γ-secalin	I	DQ2	PQT**QQPQQPFPQ**	DQ2.5-glia-γ4c/DQ8-glia-γ1a	0	1	0
928	γ-secalin	I	DQ2	PQS**QQPQQPFPQ**	DQ2.5-glia-γ4c/DQ8-glia-γ1a	0	1	0

* http://www.allergenonline.org/celiachome.shtml (Accessed on 1–3 April 2020). In the case of the glutamate residues (E) expected in-vivo by TG2-mediated deamidation, the respective sequence with unmodified glutamine (Q) residue was searched. ** The number of peptides reported in brackets refers to the analysis of the LMW-fraction, whereas all of the other counts refer to the analysis of the HMW-fraction. *** I: immunogenic; T: toxic.
